# Lactoferrin supplementation modulates the oxidative and metabolic genes by NR5A2-mediated histone modifications in deoxynivalenol-induced ileum injury

**DOI:** 10.1007/s44154-025-00242-9

**Published:** 2025-07-14

**Authors:** Xudong Guo, Xiaoyue Yuan, Zhiyong Xu, Jianhua Liu, Rongrong Lv, Yiqin Gao, Wenjing Xu, Dejun Ji, Yuting Guo

**Affiliations:** 1 Jiangsu Vocational Institute of Commerce, Nanjing, 211168 Jiangsu China; 2 Laboratory of Animal Physiology and Molecular Nutrition, Jiangsu Key Laboratory of Animal Genetic Breeding and Molecular Design, College of Animal Science and Technology, Yangzhou University, Yangzhou, 225009 China; 3 School of Nursing & School of Public Health, Yangzhou University, Yangzhou, China; 4 Institute of Hepatobiliary, Pancreatic and Spleen Diseases of Guizhou Province, Guiyang, Guizhou, 550004 China; 5 The Affiliated Hospital of Guizhou Medical University, Guiyang, Guizhou, 550004 China

**Keywords:** DON, Histone modifications, Lactoferrin, NR5 A2, Oxidative genes

## Abstract

Given that lactoferrin (LF) exerts an excellent protection of intestinal homeostasis, the underlying mechanisms, especially epigenetic regulations, are still unknown. This study aimed to investigate the effects of dietary LF epigenetically modulates the oxidative genes by histone modifications to ameliorate ileum inflammation of mice exposed to DON contaminated diet. As expected, we found in the morphology analysis that DON exposure increased ileum crypt depth (CD) and villus width (VW) but reduced villus height (VH) and VH: CD ratio compared to those of the vehicle group. Consistently, the elevated ROS and MDA, along with the decreased ATP, SOD, CAT, GSH, and complex I, III, V were observed in the DON-exposed mice ileum. In contrast, LF markedly ameliorated the impairments of morphological and biochemical indexes. Next, we conducted transcriptome analysis to explore the changed signaling pathways using the ileum RNA of the mice treated with DON or LF. Firstly, the cell cycle pathway genes were significantly downregulated in the DON-exposed mice, and LF improved the cell cycle profile. Again, gene ontology analysis showed that inflammation and oxidative stress were significantly activated by DON exposure, and these were recovered when the DON-exposed mice were supplemented with an LF diet. Consistent with these findings, the signaling pathways of the reduced oxidative phosphorylation and elevated TNFα were also observed to be ameliorated by LF treatment. Importantly, histone modifications, including acetylation, methylation, and lactylation were suggested to be the vital players involved in the DON or LF treatment, in which LF significantly increased the loss of histone modifications on these genes. With a bioinformatics analysis and validation by qRT-PCR, the nuclear receptor NR5A2 was selected as a key master in the ileum of mice stimulated by DON. LF performed the benefit function on the NR5A2-mediated oxidative stress genes *Ncoa4* and *Prdx3* in the DON-exposed mice. Moreover, a ChIP-qPCR was used to verify that histone marks involving H3K9ac, H3K18ac, H3k27ac, H3K4me1, H3K9la, and H3K18la facilitated the epigenetic regulation of NR5A2-modulated actions. We conclude that dietary LF effectively ameliorated ileum lesions induced by DON in mice by modulating oxidative genes *Ncoa4* and *Prdx3* through histone modifications.

**Supplementary Information:**

The online version contains supplementary material available at 10.1007/s44154-025-00242-9.

## Introduction

Mycotoxin contamination causes substantial economic losses due to food safety concerns and threatens human and animal intestinal health (Ayeni et al. 2023; Alshannaq et al. 2017). Deoxynivalenol (DON) is one of the most commonly detected mycotoxins in cereals and cereal by-products, and it is a fusarium-derived trichothecene mycotoxin (Eskola et al. 2020). DON destroys the intestinal barrier and induces inflammation, which in turn causes immune cells to release cytokines and leads mesenteric lymph node cells to undergo apoptosis (Gerez et al. 2015; Li et al. 2024a). In addition to affecting digestion and absorption, disruption to the intestinal barrier raises the risk of other intestinal disorders, which may have significant adverse effects on both animal and human health. In vitro and in vivo models, exposure to DON leads to inflammation and oxidative stress (Du et al. 2018; Qu et al. 2022; Li et al. 2024b). DON causes oxidative stress in a dose-dependent manner in non-malignant intestinal enterocytes (Kang et al. 2019) and colon carcinoma cells (Favero et al. 2018). DON exposure significantly increases the biomarker of lipid peroxidation malondialdehyde (MDA) production by Caco-2 cells (Wan et al. 2019). It has been reported that intestinal cell models were used to examine whether or not DON consumption can cause intestinal inflammation(Wang et al. 2023). Van De Walle et al. find that high DON intakes could induce or aggravate intestinal inflammation by causing intestinal epithelial cells to release pro-inflammatory cytokines and activating nuclear factor κB (NF-κB) (Walle et al. 2010). Recently, it has been reported that DON increases the production of tumor necrosis factor (TNF)α and releases ROS in non-cancerous intestinal epithelial cells (IEC-6) (Adesso et al. 2017). A previous study also discovers that physiologically relevant concentrations of DON can increase several pro-inflammatory cytokine mRNA levels in a jejunal epithelial cell line (Wan et al. 2013). Thus, it is necessary to determine DON's toxic consequences and mechanism to develop novel strategies for preventing and treating diseases caused by DON (Xu 2021).

Mammals'regular life activities depend on epigenetic modification, a form of gene control widespread in every animal species (Shi and Wu 2009; Guerrero et al. 2020). Histones post-translational modifications play great regulations on the interaction between DNA and nuclear proteins, including histone modifications, including methylation, acetylation, phosphorylation, ubiquitination, and lactylation (Xiao-hong et al. 2018). Notably, epigenetics has been demonstrated to be a critical field for exploring novel approaches to treating inflammatory bowel disease (Hornschuh et al. 2021). It’s worth mentioning that oxidative stress is always associated with histone modifications involving methylation and acetylation (Ebert et al. 2022). Studies have reported that DON-triggered oxidative stress is probably regulated by the histone methyltransferase MLL1 (Shi, et al. 2022). In which histone marks, including H3 K4 me3 and H3 K27ac, are implicated as the important regulator in response to the cytotoxicity and genotoxicity caused by DON (Zong et al. 2023). It is noted that oxidative stress breaks histone modifications and then modulates the key genes involved in gut health and disease. The underlying mechanisms of histone modification would provide insights into the development of therapeutic strategies for intestinal dysfunction and oxidative stress.

Lactoferrin (LF) is an iron-binding glycoprotein powerfully expressed in bovine and human milk (Li, et al. 2023). Rising indication suggests numerous physiological functions of LF, ranging from iron homeostasis and transportation to antimicrobial (Almehdar et al. 2020), anti-inflammatory (Li, et al. 2023; Actor et al. 2009), anti-apoptotic effects, and antioxidant (Blais et al. 2014). However, its exact mechanism of action is yet unidentified. As an antioxidant, LF can bind to free iron, a common catalyst for forming reactive oxygen species (ROS) that can cause oxidative stress and damage cellular components (Sies and Jones 2020). By sequestering iron, LF can reduce the Fenton reaction and the subsequent production of hydroxyl radicals, thus protecting cells from oxidative damage (Wu, et al. 2022). It has been suggested that LF is a potential epigenetic modulating factor, LF has been shown to modulate gut health and function possibly via indirect effects on histone modifications in the intestinal epithelium (Hoffman et al. 2018). Nuclear receptors (NRs) serve as sensors to regulate pathophysiologic events and drive adaptive responses via gene regulation by the assembly of transcriptional complexes with co-factors-mediated chromatin-modifying properties (Romagnolo et al. 2014). It is worth mentioning that LF has been verified to be a potential NR activator (Li et al. 2021); this evidence would support our hypothesis that LF performs gut protection by NR-mediated histone modifications. Interestingly, the motifs of nuclear receptor (NR) family factors such as NR5 A2 and RARG are overrepresented in the accessible putative regulatory elements in mouse 2–8 C embryos (Sugii and Evans 2011).NR5 A2 is also closely linked to pluripotency regulation. Nr5a2 is essential for early embryogenesis and regulates the expression of Pou5f1 in the epiblast (Labelle-Dumais, et al. 2006).

The exact mechanism of how LF interacts with mycotoxins is not explicitly detailed, given its antioxidant and anti-inflammatory properties, LF could potentially protect against the oxidative stress and inflammation induced by mycotoxins. Therefore, in the current study, we aim to investigate whether dietary LF supplementation of DON-exposed mice would enhance their resilience to ileum lesions. We also would establish that dietary LF supplementation alleviates DON-induced ileum oxidation in mice by epigenetically modulating the oxidative genes through histone modifications. Our findings will provide new theoretical perspectives for investigating how LF protects against mycotoxin-induced ileal oxidation and other adverse consequences.

## Results

### LF supplementation alleviates the ileal lesions caused by DON

**Fig. 1:**
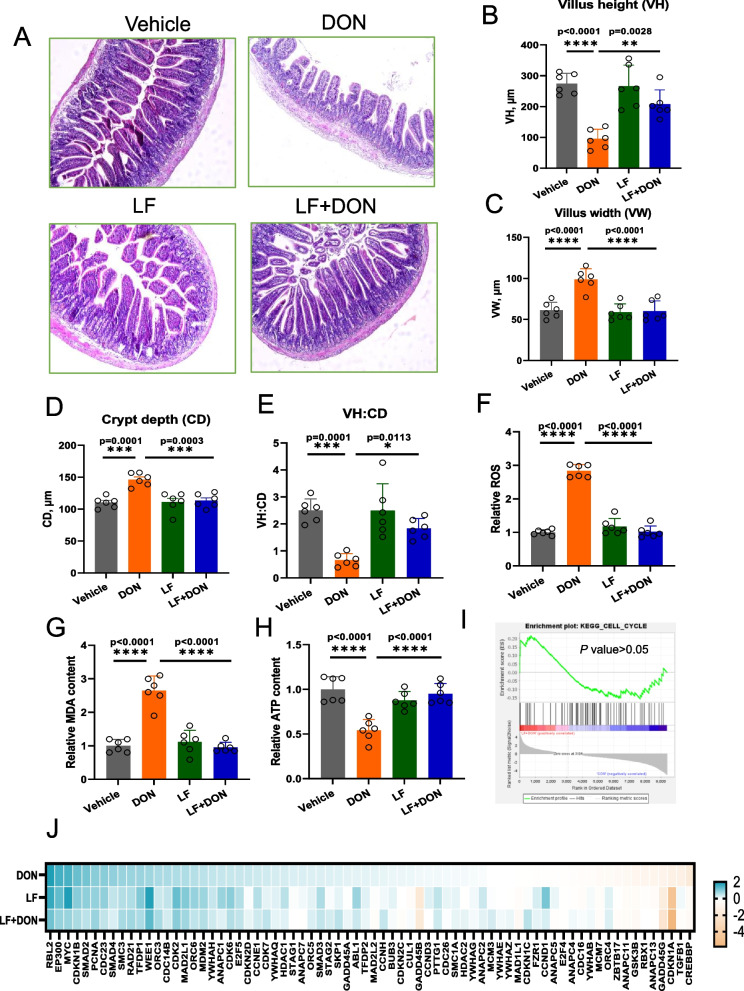
Phenotypic changes and whole-genome expression differences in ileal tissue after treatment with DON, LF, and LF + DON. **A** H&E staining sections revealed that adding LF effectively alleviates the intestinal wall and villus damage caused by DON. **B**-**E** The addition of LF reverses the differences in VH (villus height), VW (villus width), CD (crypt depth), and the VH:CD ratio caused by DON. **F**-**G** LF reduced levels of oxidative parameters ROS and MDA, and increased **H**) ATP in the DON-treated mouse ileum. **I** For the cell cycle pathway, there is no significant difference between the LF + DON and the Veh group (*P* > 0.05), reflecting its restorative effect. **J** The heatmap of pathway genes demonstrated that LF itself does not have a significant damaging effect on the ileum of mice. Values are means ± SEMs. (*)*P* < 0.05, (**) *P* < 0.01 and (***) *P* < 0.001. The circles represent the distribution of results for different samples

We first showed the phenotypic changes in ileal tissue by treating it with DON, LF, and LF + DON in the mice. Using the H&E staining sections as shown in Fig. 1A-E, the results revealed that the addition of LF effectively alleviates intestinal villus damage, including the increased villus width, crypt depth, and the reduced villus height and VH to CD ratio caused by DON exposure. In agreement with the morphological changes, oxidative parameters like ROS and MDA were significantly elevated in the DON-treated mouse ileum. In this model, LF markedly reduced the levels of ROS and MDA, as shown in Figs. 1F and G. Again, the ATP was also observed to be increased when the DON-exposed mice were treated with LF. In the above statistics, both the vehicle group and the DON group show extremely significant differences, while the combined treatment group has a significant restorative effect compared to the DON group. With a bioinformatics analysis using transcriptome analysis, the GSEA data revealed no significant difference between the LF + DON and the Veh groups in the cell cycle pathway (Fig. 1I), reflecting its restorative effect of LF. Consistently, the heatmap of pathway genes demonstrated that LF itself has no significant impact on the ileum of mice (Fig. 1J). The results indicated that LF supplementation in mice could effectively alleviate the ileal lesions caused by DON.

### LF enhances anti-oxidation in the DON-stimulated ileum

**Fig. 2:**
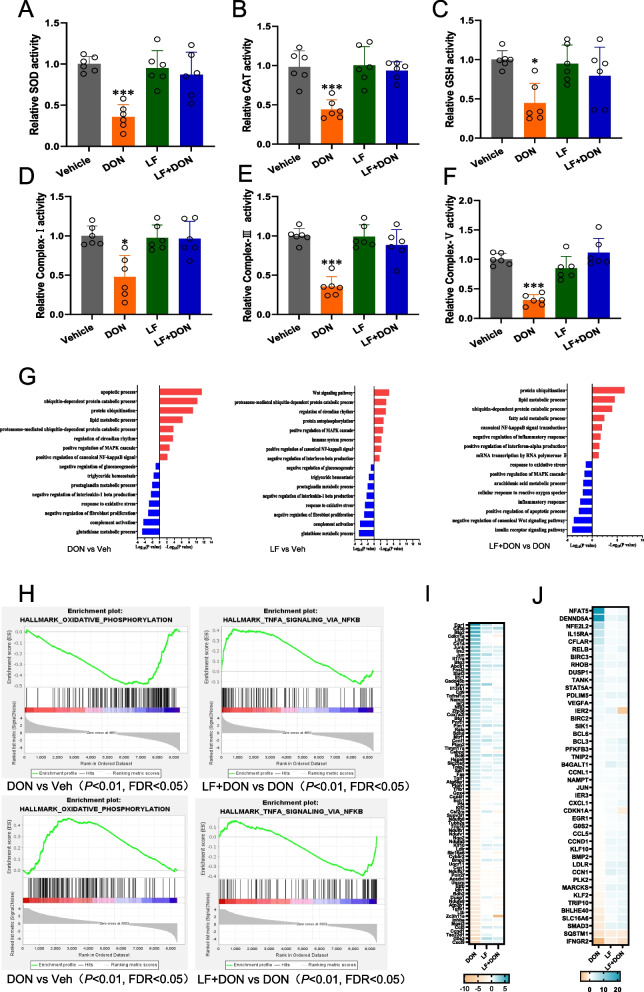
Anti-oxidation parameters and Gene expression data obtained from RNA-seq were subjected to pathway enrichment analysis, primarily focusing on pathways related to inflammation, immunity, and oxidative stress. **A**-**F** Anti-oxidation parameters SOD, CAT, GSH, complex-I, III, V were all decreased after DON exposure and were activated by LF treatment. **G** KEGG enrichment analysis revealed that compared to the Veh group, DON treatment significantly upregulated processes such as inflammation, oxidative stress, and apoptosis, while the combined treatment significantly downregulated these pathways, and the sole LF treatment did not have a particularly significant impact on these pathways. **H** Oxidative phosphorylation pathway was significantly downregulated by DON treatment, while the TNFα signaling pathway was significantly upregulated, and **I**-**J**) plotted a heatmap of their key genes. Values are means ± SEMs. (*) *P* < 0.05 and (***) *P* < 0.001. The circles represent the distribution of results for different samples

In parallel to the increased ROS and MDA in the DON-exposed mouse ileum, the key parameters of anti-oxidation involving SOD, CAT, GSH, complex-I, III, V were all remarkedly decreased in response to DON exposure. These decreased anti-oxidative enzymes were activated by treatment of DON group with LF (Fig. 2A-F) Compared to DON and vehicle groups. Moreover, the RNA-seq analysis was performed to delineate DON's effects on the ileum's core transcription programs. In the pathway enrichment analysis of differential genes between the DON group and the Veh group, the LF group and the Veh group, and the LF + DON group and the DON group. It was found that compared to the Veh group, the DON group significantly upregulated pathways related to inflammation, oxidative stress, immune regulation, and apoptosis. The combined treatment significantly downregulated these pathways. However, the LF treatment alone did not significantly impact these pathways (Fig. 2G). GSEA also confirmed that DON and LF administration significantly altered this fluctuation in the inflammatory response pathway in the ileum of the DON + LF group compared to the DON group, a key marker related to immune and oxidative stress. It was observed that LF could reverse the activation or inhibition of relevant pathways caused by DON (Fig. 2H). The oxidative phosphorylation pathway was significantly downregulated by DON treatment, while the TNFα signaling pathway was upregulated considerably and plotted a heatmap of their key genes (Fig. 2I-J). The above results indicated that the supplementation with LF effectively alleviated DON-induced oxidative stress at the transcriptional levels.

### Histone modifications are potential factors in response to DON or LF treatment

**Fig. 3:**
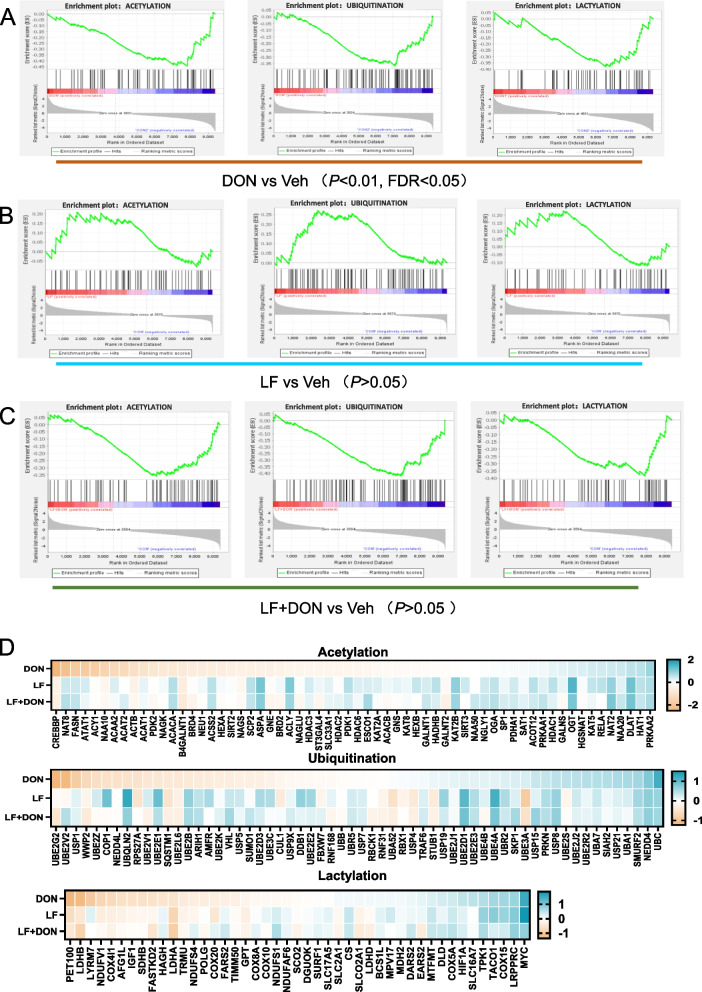
Compared to the vehicle group, DON treatment significantly downregulated pathways related to acetylation, ubiquitination, and lactylation modifications, while LF treatment alone and combined LF + DON treatment showed no significant difference compared to the vehicle group. **A**-**C** GSEA-generated enrichment profiles of three epigenetic modification-related pathways and **D**) the corresponding heatmaps of key gene expressions

Given the importance of epigenetic regulations in the regulation of oxidative genes, we further the disturbance pathways identified by gene ontology analysis. The results showed several pathways downregulated (*P* < 0.05) regarding acetylation, ubiquitylation, and lactylation when dietary DON was added to the mice compared to that of the Veh group (Fig. 3A). However, LF treatment alone and combined treatment of LF + DON had no significant effect on these pathways (*P* > 0.05) (Fig. 3B-C). Moreover, the pathway-focused data showed that the critical genes involved in the histone modifications were significantly down-regulated by DON exposure. In contrast, LF treatment alone or the combination of LF and DON significantly increased these gene expressions (Fig. 3D). The above results indicated that supplementation with LF supplementation played critical roles in histone acetylation, ubiquitination, and lactylation that would be benefit for anti-oxidation by epigenetic regulations in DON-treated mice.

### Nuclear receptor NR5 A2 epigenetically modulates the transcriptional suppression of Ncoa4 and Prdx3

**Fig. 4:**
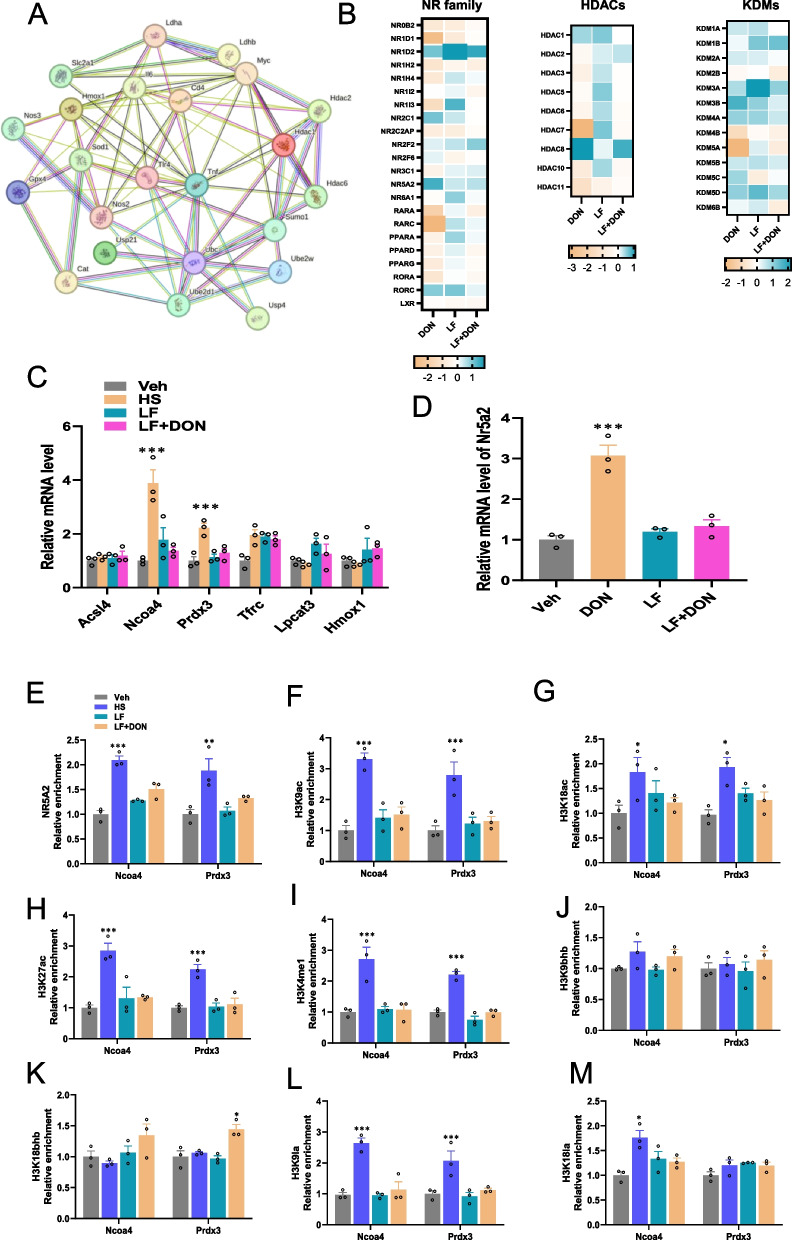
This figure illustrates the regulatory role of nuclear receptor NR5 A2 and histone modifications in controlling the expression of *Ncoa4* and *Prdx3* in mice exposed to DON and treated with LF, as well as the enrichment and changes in related histone marks upon DON stimulation. **A** DON exposure modifies histone modification at the locus of *NCOA4* and *PRDX3*. A) STRING analysis of protein–protein interactions related to inflammatory, oxidative stress, and epigenetic modification-associated proteins. **B** Heatmap of genes from the nuclear receptor, HDAC (Histone Deacetylase) family, and KDM (Lysine Demethylase) family. qRT-PCR detects the gene expression of **C**) oxidative genes and **D**) NR5 A2. ChIP-qPCR reveals the relative enrichment of **E**) NR5 A2 and histone marks **F**) H3 K9ac. **G** H3 K18ac. **H** H3 K27ac. **I** H3 K4 me1. **J** H3 K9bhb. **K** H3 K18bhb. **L** H3 K9 la. **M** H3 K18 la at the locus of *Ncoa4* and *Prdx3*. (*) *P* < 0.05 and (***) *P* < 0.001 compared with the uninfected sample. The circles represent the distribution of results for different samples

We next performed a STRING analysis to explore the protein–protein interactions related to inflammatory, oxidative stress, and epigenetic modification-associated proteins (Fig. 4A). The results exhibited that histone deacetylases (HDACs) are closely associated with key anti-oxidative and inflammatory genes, including *Gpx4*, *Tnf*, *Cat*, *Cd4*, and *Il6*. Given that nuclear receptors exert pivotal actions on oxidative genes through histone modifications, we deeply evaluated the individual gene expression patterns in the DON-exposed ileum with or without LF treatment. In this process, we found that NR5 A2 is the key candidate, along with histone modifications that dominate the antioxidative gene transcripts (Fig. 4B). Moreover, we used a qRT-PCR analysis to validate the oxidative gene changes in the DON-exposed mice with or without LF treatment. Consistent with the transcriptomic results, the genes *Ncoa4* and *Prdx3* were significantly up-regulated by DON exposure (Fig. 4C). These two increased oxidative genes were markedly inhibited by LF supplementation. For the validation of the *Nr5a2* gene mRNA level, we also found a similar pattern, where *Nr5a2* was highly expressed in the DON group but was reduced to the control level with an LF treatment (Fig. 4D). It suggests that the changed genes of *Ncoa4* and *Prdx3* were modulated by NR5 A2 and histone modifications. We then performed ChIP-qPCR to detect the NR5 A2 and transcriptional activation-linked histone marks H3 K9ac, H3 K18ac, H3 K27ac, H3 K4 me1, H3 K9bhb, H3 K18bhb, H3 K9 la, and K18 la at the locus of *Ncoa4* and *Prdx3* in the ileum. As expected, NR5 A2 enrichment was dramatically enhanced by DON stimulation as shown in Fig. 4E. However, LF treatment remarkably decreased the enrichments compared to that of the vehicle group. Notably, histone marks including H3 K9ac, H3 K18ac, H3 K27ac, H3 K4 me1, and H3k9 la related to transcriptional activation were significantly enriched by DON at the developers of *Ncoa4* and *Prdx3* compared to Veh group, respectively. While the supplementation of the dietary LF significantly (*P* < 0.05) reduced their enrichments compared to the DON group (Fig. 4F-I and L). Additionally, enrichment of K18 la at target loci of *Ncoa4* significantly increased in the DON compared to the Veh group but did not affect *Prdx3* (Fig. 4M). However, the occupancies of H3 K9bhb and H3 K18bhb were not significantly changed at the promoters of *Ncoa4* and *Prdx3* (Fig. 4J, K). These findings demonstrated the important roles played by nuclear receptor *Nr5a2* and histone modifications in the regulation of *Ncoa4* and *Prdx3* in mice exposed to DON and treated by LF.

## Discussion

The intestinal epithelium, especially in the small intestine, exhibits a characteristic structure when exposed to DON (Li, et al. 2022). DON-contaminated diets had been linked to altered gut morphology in monogastric animals, as evidenced by several studies (Xue et al. 2023). These effects include villi atrophy and fusion, reduced crypt depth, fewer goblet cells, increased lymphocyte abundance, widespread apical necrosis, and a decreased villi-to-crypt ratio in the ileum (Song et al. 2022; Wang, et al. 2018). These are in line with our study, in which we found that DON exposure caused a decrease in ileum villus height and villus height to crypt depth and an increase in crypt depth and villus width in mice. Yu et al. (Yu et al. 2011) reported that the gut capacity for digestion is directly related to the height of the intestinal villi. The presence of the oedema in the lamina propria and cytoplasmic vacuolation of enterocytes in the ileum in animals given DON. The study showed that ileum villus height and villus height to crypt depth increased, and crypt depth and villus width decreased with LF supplementation in DON mice. Similarly, Lactoferrin supplementation leads to increased villus height and villus height to crypt depth ratio and decreased crypt depth and villus width, compared with the same measurements in DON-fed mice (Song et al. 2022) and piglets (Xiao, et al. 2013).

Oxidative stress is caused by an imbalance between the production of reactive oxygen species and antioxidant defenses (Halliwell 2007). Few studies focused on the oxidative stress that DON induced in mice intestines. Extracellular or intracellular signals activate a sophisticated machinery that sets up a series of processes that result in cell lysis and the breakdown of nuclear DNA, the ultimate goal of apoptosis. DNA damage, tumor necrosis factor (TNF) or glucocorticoids production, and Fas/Apo-1 activation are joint triggering factors (Santavanond et al. 2021). Oxidative phosphorylation dominates ATP generation. The inner mitochondrial membrane is home to oxidative phosphorylation complexes, which receive high-energy electrons from NADH (which is generated when acetyl-CoA is oxidized) (Vercellino and Sazanov 2022). Before reaching the final electron acceptor, molecular oxygen, these electrons are transferred through various oxidative phosphorylation complexes that include heme, copper iron sulfur groups, and flavins as electron transporters (Wilson 2017). Prevention of inflammatory disease and tissue homeostasis preservation depends on optimal TNF signaling regulation (Quickelberghe et al. 2018). TNF receptor 1 (TNFR1) mediates the majority of the inflammatory effects of TNF. TNF antagonists, neutralizing TNFR1-NF-κB signaling, positively affect inflammatory and autoimmune diseases (Varfolomeev and Vucic 2018). In the present study, DON exposure induced oxidative stress in the ileum, whereas the treatments with LF were able to keep their levels similar to the vehicle group. To the author's knowledge, no previous data evaluating inflammation, oxidative stress, immune regulation, apoptosis, or modulation by DON in the ileum exists. Our findings are consistent with existing results. DON has been proven in both in vivo and in vitro experiments to induce tissue inflammation and exacerbate inflammatory damage in infection models by activating the NF-κB signaling pathway and upregulating pro-inflammatory factors such as TNF-α, IL-6, and IL-1β (Tardivel et al. 2015; Pierron, et al. 2024). Additionally, DON can promote excessive generation of mitochondrial ROS, inhibit the activity of antioxidant enzymes such as SOD and GSH-Px, leading to cellular oxidative stress and DNA damage (Mishra et al. 2014). In contrast, LF as a natural iron-binding immunomodulatory molecule, can reduce ROS generation and downregulate the levels of pro-inflammatory cytokines. It also helps maintain iron homeostasis and tissue barrier function, providing dual protection against inflammation and oxidative damage (Park et al. 2017; Berthon et al. 2022).

Nuclear receptors are essential for many physiological processes, including growth and metabolism, proliferation, survival, and apoptosis (Fuchs et al. 2022). Consequently, Nuclear receptor dysfunctions have been closely linked to a wide range of diseases, including diabetes, obesity, infertility, inflammation, cardiovascular disorders, and prostate and breast cancers (Fuchs et al. 2022; Ye et al. 2024). The regulatory molecules known as nuclear receptors regulate cellular signals by interacting with specific DNA sequences. The NR5 A subfamily, which consists of four members (Nr5a1–Nr5a4), includes NR5 A2. NR5 A2 is implicated in a variety of biological processes, including steroidogenesis, embryonic stem cell pluripotency, reverse cholesterol transfer, embryonic development and differentiation, and adult homeostasis. It has been shown that NR5 A2 haploinsufficiency is linked to gastrointestinal, pancreatic, and chronic pancreatitis (Sandhu et al. 2021). Our current study, NR5 A2, significantly increases DON exposure at the developers of *Ncoa4* and *Prdx3* and demonstrated the potential of LF as a strategy to improve intestinal expression against DON challenges.

Histone modifications are highly sensitive to environmental stimuli such as oxidative stress and play an important role in regulating chromatin structure and gene expression (Maunakea et al. 2010). H3 K9ac and H3 K4 me1 are epigenetic modifications that affect the histone H3 protein and are related to the activation of gene transcription (Ji et al. 2019). Histone modification targeting is a new cancer therapeutic approach for ileum injury (Ramaiah et al. 2021). We found that DON exposure dramatically elevated the occupancy of these two histone marks, supporting the notion that H3 K4 me1 and H3 K9ac are key regulators of gene regulation (Gandhi, et al. 2022). In this study, mice models of the histone marks H3 K9ac, H3 K4 me1, and H3k9 la related to transcriptional activation were significantly up-regulated by DON. Recent research has revealed a unique mechanism for transmitting cellular metabolic patterns to gene expression programs by activating or inhibiting gene transcription H3k9 la (Zhang et al. 2019), a lactate-dependent epigenetic modification. Several biological processes are affected by acetylation, such as macrophage M1/2 polarization, and an increase in microbial dysfunction (Pan, et al. 2022). Our results showed that H3 K9 la was highly enriched in gene promoters and correlated with the activity of genes associated with *Ncoa4* and *Prdx3*. The role of H3 K18ac and H3 K27ac in tissue damage is gradually being recognized. Increased levels of H3 K18ac and H3 K27ac were found to promote acute tissue damage by activating inflammation and macrophage polarization, and an inhibitor of the histone acetyltransferase p300/CBP was shown to correct this epigenetic damage (Ma, et al. 2023). Gradually, the importance of H3 K18ac and H3 K27ac in disorders is becoming clear. Elevated levels of H3 K18ac were shown to exacerbate acute tissue injury by inducing inflammation and polarization of macrophages. Conversely, an inhibitor of the histone acetyltransferase p300/CBP was shown to alleviate acute tissue injury by correcting this epigenetic degradation (Peng et al. 2019). Unlike the abnormal elevation of H3 K18ac and H3 K27ac in the present studies, we found that the increase in H3 K18ac and H3 K27ac of *Ncoa4* and *Prdx3* was the primary differential mode in response to ileum tissue damage induced by DON exposure. These results suggest that the disruption mode of H3 K18ac and H3 K27ac may be complex in response to different types of ileum injury. The observation in this study will require further experimental investigation to better inform the overall role of H3 K18ac, H3 K27ac, H3 K9ac, H3 K4 me1, and H3k9 la as determinants of oxidative stress.

In summary, the findings of this study demonstrated that dietary LF plays a protective role against DON-induced ileum lesions in mice by epigenetically modulating oxidative gene expression through histone modifications. LF exhibited potential as a therapeutic agent to counteract the detrimental effects of DON on gut health by reducing the histone marks associated with transcriptional activation of the *Ncoa4* and *Prdx3* genes and the expression of key oxidative genes. These results highlighted the importance of investigating dietary components such as LF for their protective effects against mycotoxin-induced oxidative damage. They provided a foundation for further research into dietary interventions to enhance food safety and improve overall health outcomes in humans and animals. As a natural endogenous substance, lactoferrin is non-toxic and harmless. Simply by being used as a feed additive, it can greatly alleviate intestinal damage in animals. Studies had also confirmed that LF helps stabilize the gut microbiota, inhibits the colonization and proliferation of intestinal pathogens, and promotes the growth of probiotic strains(Vega-Bautista, et al. 2019). It can be seen that LF is expected to play an important role in the intestinal health of animals and even humans in the future.

## Methods and materials

All procedures were conducted in accordance with the ARRIVE (https://arriveguidelines.org) guidelines. The animal study mentioned in this article has been approved by the Institutional Animal Care and Use Committee (IACUC) of the Animal Experimental Ethics Committee of Yangzhou University and conducted under the authority of the Project License: SYXK(Su)2022–0044, issued by the Department of Science and Technology of Jiangsu Province. The method of euthanasia for the animals was cervical dislocation after ensuring anesthesia by intraperitoneal injection of barbiturates and barbituric acid derivatives.

### DON production and analysis

Fusarium graminearum strain W3008 was kindly provided by the College of Animal Science and Technology, Yangzhou University, China. The strain was cultured on potato dextrose agar at 28 °C for seven days to obtain mature spores. Three hundred grams of maize, fifty grams of rice, and 140 mL of sterilized distilled water were added to a 1-L conical flask and then autoclaved at 121 °C for 20 min. Each flask was inoculated with F. graminearum at 1 × 10^6^ spores/g and incubated at 28 °C and 85% humidity for 28 days. Finally, the mold-contaminated sample in each flask was dried in an air oven at 65 °C overnight, mixed, and sampled to determine the DON content. The resulting coated product was confirmed to contain approximately 300 mg/kg DON. DON content was determined using the AgraQuant® DON ELISA test kit following the manufacturer's protocol (Romer Labs, Singapore).

### Animals and Experimental Design

Approved by the Institutional Animal Care and Utilization Committee (IACUC) of the Animal Experimental Ethics Committee of Yangzhou University, the study was conducted in accordance with the local legislation and institutional requirements. Twenty-four male BALB/c mice (aged 5 weeks, 24.7 ± 0.32 g) were acclimatized to a commercial diet (Anlimao, Jiangsu, China) as the basal diet for a 5-day adaptation. Then they were randomly divided into 4 groups (6 mice per group, 2 mice per cage): Vehicle group (basal diet, n = 6); Deoxynivalenol (DON) group (basal diet supplemented with 12 mg/kg DON, n = 6); Lactoferrin (LF) group (basal diet with oral administration of LF at 10 mg per day n = 6); Deoxynivalenol + Lactoferrin (DON + LF) group (basal diet with oral administration of LF at 10 mg per day + 12 mg/kg DON, n = 6) based on previous study (Li, et al. 2023). Mice cages were equipped with a one-side self-feeder and a nipple water-feeder for libitum access to feed and water and raised in a room at constant temperature (25 ± 2 °C) and humidity (50% ± 10%) with a 12-h light–dark cycle throughout the experiment. On the week 6, the mice were subjected to cervical dislocation after being ensured anesthesia by intraperitoneal injection of barbiturates and barbituric acid derivatives. Ileum tissue and contents were collected.

### Hematoxylin and eosin staining

Hematoxylin and eosin staining of the intestine was performed. Ileum sections were fixed in a solution of 4% paraformaldehyde. Sections were first exposed to xylene and ethanol solutions for 15 and 5 min, respectively. After that, they were stained with hematoxylin for 5 min, washed with water for 5 min, and then stained with eosin solution for 1–3 min. Then, it was cleaned with ethanol and sealed. Well-oriented villus-crypt units were chosen in replicate, with a total of six replicates (18 measurements per sample). A 10 × objective lens was used to view the sections, and an Olympus microscope (U-TV0.63XC, Tokyo, Japan) was used to capture pictures.). Using Infinity Analyze software (Version 7, Lumenera Corporation, Ottawa, ON, Canada), various ileum morphological parameters were measured, including villus height (VH) and width (VW) distances from the villus tip to the crypt, crypt depth (CD) distances from the villus base to the submucosa, and the ratio of villus height to crypt depth (VH/CD).

### Biochemical measurements

Ileum tissues were first homogenized and then washed in PBS, after which the supernatant was collected. The contents of ROS, MDA, catalase (CAT), glutathione (GSH), superoxide dismutase (SOD), and the activities of mitochondrial respiratory chain complexes I, III, and V were measured using appropriate commercial assay kits in the ileum and investigated. Commercial assay kits include Nanjing Jiancheng Institute of Bioengineering (Nanjing, China), OxySelect In Vitro ROS/RNS Assay Kit (Cell Biolabs, STA-347, San Diego, CA, USA), and appropriate commercial assay kits from Comin Technologies, Co., Ltd., Suzhou, China. All procedures were performed following the kit’s protocol.

### Real-time quantitative PCR

RNA was extracted from the ileum of mice from four distinct groups (6 mice per group), using Trizol (Invitrogen, Waltham, MA, USA) according to the manufacturer's instructions and stored at −80 °C. The quantity and purity of the extracted RNA were assessed via a protein-nucleic acid analysis instrument (ND-2000UV, Thermo Fisher, Waltham, USA) and confirmed through 1% agarose gel electrophoresis. Subsequently, the RNA was converted into cDNA using the transcript All-in-One First-Strand cDNA Synthesis Super MIX for qPCR (QIAGEN, Frankfurt, Germany). The reverse transcription mixture consisted of: 0.5 μg of total RNA, 5 μL of 5 × TransScript All-in-one SuperMix for qPCR, 0.5 μL of gDNA Remover, and nuclease-free H2O was adjusted to a total volume of 10 μL. The reverse transcription was carried out at 42 °C for 15 min, followed by 85 °C for 5 s. Post-transcription, 90 μL of nuclease-free H2O was added to the mixture, which was then stored at −20 °C. Real-time PCR was performed using a LightCycler® 480 IIReal-time PCR Instrument (Roche, Basel, Switzerland) with a PCR efficiency ranging from 94 to 105%. The PCR reaction mixture (10 μL) included 1 μL of cDNA, 5 μL of 2 × PerfectStartTM Green qPCR SuperMix, 0.2 μL of forward primer, 0.2 μL of reverse primer, and 3.6 μL of nuclease-free water. The reactions were conducted in 384-well optical plates (Roche, Basel, Switzerland) with an initial denaturation at 94 °C for 30 s, followed by 45 cycles of 94 °C for 5 s and 60 °C for 30 s. A melting curve analysis was performed post-PCR to ensure the specificity of the PCR product. Each sample was analyzed in triplicate. Additionally, qRT-PCR was conducted using an ABI StepOne Plus Real-Time PCR System (Applied Biosystems, CA, USA) with AceQ® qPCR SYBR Green Master Mix (Vazyme, Nanjing, China). The mRNA expression levels were normalized to glyceraldehyde-3-phosphate dehydrogenase (GAPDH) and quantified using the 2^−ΔΔCt^ method. Primers’ sequences information is listed in Supplementary table S1.

### RNA-Seq and analysis

RNA was extracted from the ileum of mice from four distinct groups (6 mice per group), using Trizol (Invitrogen, Waltham, MA, USA). The integrity and quality of the extracted RNA were then assessed utilizing the Agilent Bioanalyzer 2100 system (Agilent Technologies, CA, USA). For the preparation of the RNA-seq libraries, the Ultima Dual-mode mRNA Library Prep Kit (Yeasen, China) was employed.

Subsequently, these libraries underwent comprehensive sequencing with an Illumina HiSeq 2000 sequencer at Kidio Biotech Ltd (Guangzhou, China). This sequencing process strictly adhered to the protocols specified by the manufacturer. The resulting raw data were aligned against the GRCm39 reference for mouse gene expression, which incluÞs both the gff3 dataset and the genomic fasta dataset, originally sourced from the Ensembl database.

Raw data were processed using Trim Galore v0.6.5 (Babraham Bioinformatics-Trim Galore!), STAR-v2.7.10b (GitHub-alexdobin/STAR: RNA-seq aligner) and rsem-v1.3.3 (https://deweylab.github.io/RSEM/) to obtain the FPKM values for further analysis. Gene Set Enrichment Analysis (GSEA 4.1.0) software was used to identify GO terms enriched in differentially expressed genes (DEGs). Moreover, pathways of statistically enriched biological processes or DEGs were ranked and classified by the Metascape database (http://metascape.org/) for GO and KEGG pathways.

### ChIP-qPCR measurement

Ileal tissues were cut into small pieces, fixed in 1% formaldehyde for five minutes, and then fixed with ice-cold glycine for five minutes. After that, the samples were resuspended in HEPES lysis buffer containing the following components: pH 8.0, 140 mM NaCl, 1 mM EDTA, 10% glycerol, 0.5% NP-40, and 0.25% Triton X-100. Samples were suspended in cutting buffer (pH 8.0, 0.1% SDS, 1 mM EDTA, and 10 mM Tris–HCl) and submitted to sonication using a Covaris E220, as directed by the manufacturer.

Crude chromatin fragments were incubated with designated antibodies overnight at 4 °C and then treated with protein-G magnetic beads. The following step in library generation, ChIP-qPCR, requires purified ChIP-DNA. Chromatin immunoprecipitation was performed as previously described (Liu, et al. 2021; Li, et al. 2021), with the following modifications. Magnetic beads (Thermofisher Scientific, 10004D, Waltham, MA, USA) were first treated with immune serum for two hours at 4 °C, and then binding occurred to eliminate coarse chromatin extracts from ileal tissue. The pre-treatment chromatin solutions were incubated for an additional night at 4 °C using the specified antibodies. Then, BSA was used to block the protein A beads, and sonicated salmon sperm DNA was added to precipitate the samples. Previous ChIP preparations achieved immunoprecipitated complexes by eluting, briefly vertexing, and diluting the 20 mM dithiothreitol solution for 0.5 h at 37 °C. Samples were incubated overnight at 4 °C with the indicated antibodies for secondary ChIP, and ChIP-ed DNA was subsequently analyzed using qRT-PCR.

### Statistical analysis

A completely randomized design was used to apply statistical analysis to all data. The GLM technique was followed in this task using GraphPad Prism version 9.0 software (GraphPad Software, San Diego, CA, USA). One-way analysis of variance (ANOVA) was used to evaluate the data. Statistical difference was acceptable when *P* < 0.05. Data were shown as mean ± standard error (SEM).

## Conclusions

The findings of this study demonstrate that dietary LF plays a protective role against DON-induced ileum inflammation in mice by epigenetically modulating oxidative gene expression through histone modifications. LF exhibits potential as a therapeutic agent to counteract the detrimental effects of DON on gut health by reducing the histone marks associated with transcriptional activation of the *Ncoa4* and *Prdx3* genes and the expression of key oxidative genes. These results highlight the importance of investigating dietary components such as LF for their protective effects against mycotoxin-induced inflammation. They provide a foundation for further research into dietary interventions to enhance food safety and improve overall health outcomes in humans and animals.

## Supplementary Information

Supplementary Material 1.

## Data Availability

The original transcriptome data proposed in this study has been preserved in the National Center for Biotechnology Information (https://www.ncbi.nlm.nih.gov/), the preservation number is PRJNA1142198.

## Abbreviations

ATP: Adenosine Triphosphate
CD: Crypt Depth
ChIP: Chromatin Immunoprecipitation
ChIP-qPCR: Chromatin Immunoprecipitation-Quantitative Polymerase Chain Reaction)
CAT: Catalase
DON: Deoxynivalenol
GSH: Glutathione
H&E: Hematoxylin and Eosin
IACUC: Institutional Animal Care and Use Committee
KEGG: Kyoto Encyclopedia of Genes and Genomes
LF: Lactoferrin
MDA: Malondialdehyde
Ncoa4: Nuclear Receptor Coactivator 4
NR5 A2: Nuclear Receptor Subfamily 5 Group A Member 2
Prdx3: Peroxiredoxin 3
qRT-PCR: Quantitative Real-Time Polymerase Chain Reaction
RNA-seq: RNA Sequencing
ROS: Reactive Oxygen Species
SOD: Superoxide Dismutase
TNFα: Tumor Necrosis Factor Alpha
VH: Villus Height
VW: Villus Width
