# Supplementary material for: Lactoferrin supplementation modulates the oxidative and metabolic genes by NR5A2-mediated histone modifications in deoxynivalenol-induced ileum injury
Source: Stress Biol. 2025 Jul 14;5(1):47. doi: 10.1007/s44154-025-00242-9 (PMC12259518; doi:10.1007/s44154-025-00242-9)
Supplement: Supplementary file 1 — Supplementary Material 1. [file 44154_2025_242_MOESM1_ESM.docx]

Table 1. The primers sequences list

|  | |
| --- | --- |
| **Gene name** | **Primer Sequence (5’to3’)** |
| Acsl4 | Forward: CAATAGAGCAGAGTACCCTGAG |
|  | Reverse: TAGAACCACTGGTGTACATGAC |
| Ncoa4 | Forward: GGAGCTTGCTATTGGTGGAGTAC |
|  | Reverse: TAGATGAGATCGACCTGTTCATTG |
| Prdx3 | Forward: TCAGTGGATTCCCACTTCAGTCA |
|  | Reverse: TCCGTAGTCTCGGGGATATCTGCT |
| Tfrc | Forward: TCACACTCTCTCAGCTTTAGTG |
|  | Reverse: TGGTTTCTGAAGAGGGTTTCAT |
| Lpcat3 | Forward: TCACACTCTCTCAGCTTTAGTG  Reverse: TGGTTTCTGAAGAGGGTTTCAT |
| Hmox1 | Forward: GAGACGCTTTACATAGTGCTGT |
|  | Reverse: AAGTTGATAACCGAGTCGTTCT |
| GAPDH | Forward: ACATCATCCCTGCTTCTACTGG |
|  | Reverse: CTCGGACGCCTGCTTCAC |
